# Bilateral acute angle-closure glaucoma following tramadol subcutaneous administration

**DOI:** 10.1186/s12886-018-0715-4

**Published:** 2018-02-17

**Authors:** Anis Mahmoud, Fatma Abid, Imen Ksiaa, Sourour Zina, Riadh Messaoud, Moncef Khairallah

**Affiliations:** 1 Department of Ophthalmology, Tahar Sfar University Hospital, Mahdia; Faculty of Medicine, University of Monastir, Monastir, Tunisia; 2 Department of Ophthalmology, Fattouma Bourguiba University Hospital, Faculty of Medicine, University of Monastir, Monastir, Tunisia

## Abstract

**Background:**

To report a case of bilateral acute angle closure-glaucoma following the use of subcutaneous Tramadol.

**Case presentation:**

A 42-year-old healthy man with unremarkable past medical and ocular history, was admitted to the Orthopedic Department for surgical treatment of a bilateral open fracture of the femur following a road accident. Three hoursafterTramadolsubcutaneous injection, the patient complained of a bilateral acute painful visual loss with persistent vomiting. An ocular examination showed bilateral acute angle-closure-glaucoma. The patient was treated with topical anti-glaucoma therapy and intravenous Mannitol 20%.After resolution of ocular hypertension attack, NdYag laser peripheral iridotomy was performed on both eyes. After a follow-up period of 7 days visual acuity improved to 20/20 in both eyes and intraocular pressure returned to normal levels.

**Conclusions:**

This case highlights the risk of developing bilateral acute angle-closure glaucoma after Tramadol administration.

**Keywords:**

Angle closure glaucomaMydriasisTramadolAnterior segment OCT

## Background

Acute angle-closure glaucoma (AACG) is one of the most vision-threatening conditions being the leading cause of bilateral blindness in the Asian population [1].

Several types of drugs may precipitate bilateral AACG such as adrenergic, anticholinergic [2] and sulfonamide-derived medications (diuretic, antibiotics, antiepileptic and antidepressant) [3, 4].

Herein, we report a case of bilateral AACG following Tramadol subcutaneous administration.

## Methods

It was a single case report. Written informed consent was obtained from the patient before publishing.

## Case presentation

A 42-year-old healthy male was admitted to the Emergency Department after a road accident. He was referred to the Orthopedic Department for management of a bilateral open fracture of the femur.

To deal with acute pain, the patient received a subcutaneous injection of 100 mg of Tramadol after which he experienced a transient bilateral blurred vision that resolved spontaneously. Eight hours later, a second subcutaneous injection of 100 mg of Tramadol was administered and 3 h later, the patient started complaining of severe blurred vision with headache and vomiting.

**Fig. 1:**
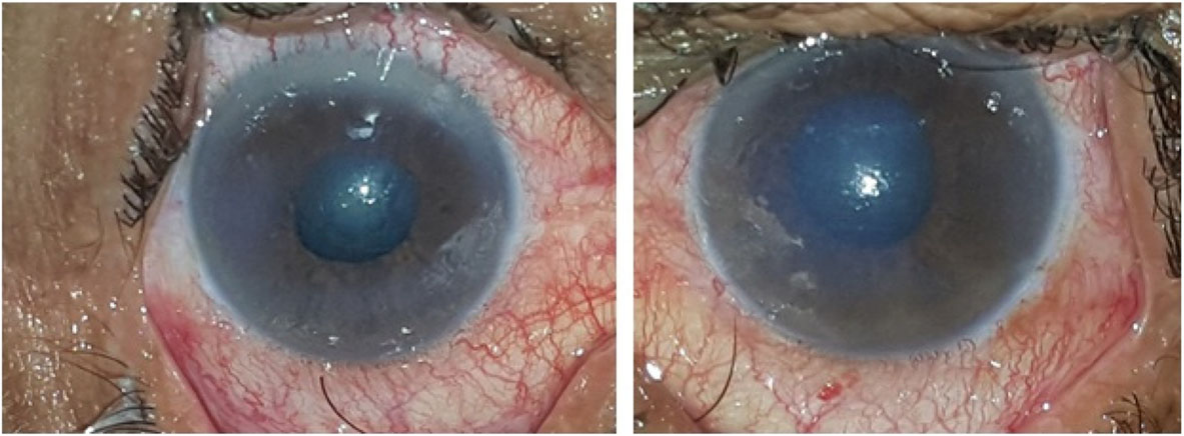
Slit-lamp photograph showing conjunctival injection associated with corneal edema and mid-dilated pupil in both eyes

**Fig. 2:**
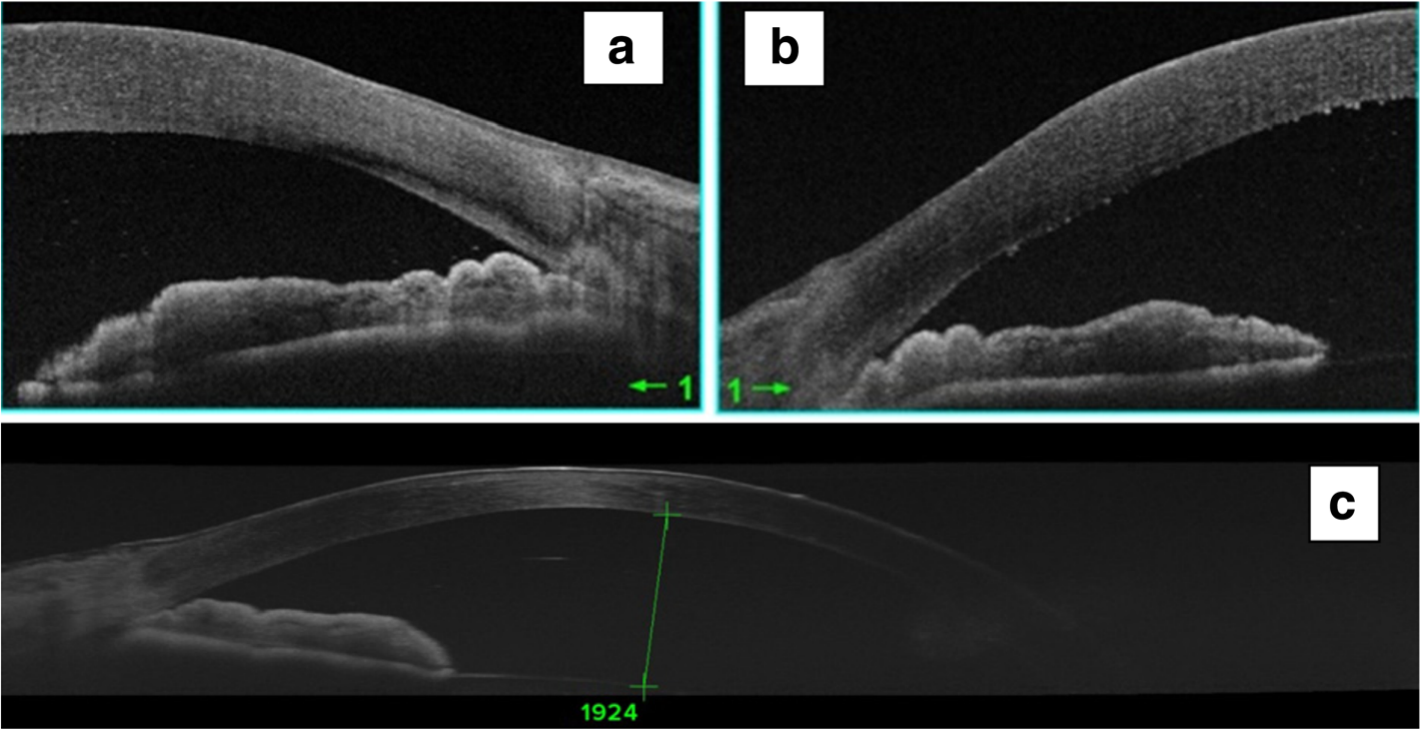
AS-OCT performed at the acute phaseconfirms the angle closure in both eyes **(a** and **b)**. Note the presence of shallow anterior chamber **(c)**

On examination,the visual acuity was 20/400 in the right eye (RE) andwas limited to light perception in the left eye (LE). A slit lamp examination showed bilateral conjunctival injection associated with corneal edema. The anterior chambers were narrow and the pupils were mid-dilated with a sluggish light reaction in both eyes (Fig. 1). The intraocular pressure (IOP) with applanation tonometry was 46 mmHg in the RE and 55 mmHg in the LE. A gonioscopy showed a narrow angle with no visible structures. The fundus details were hidden by the corneal edema. An anterior segment optical coherence tomography (AS-OCT) performed during the acute phase demonstrated a closed iridocorneal angle with a markedly reduced anterior chamber depth of 1.9 mm (Fig. 2).

The diagnosis of bilateral drug-induced AACG in a predisposed patient was therefore made. The suspected culprit drug, Tramadol, was discontinued and intravenous mannitol 20%, topical anti-glaucoma drops and pilocarpin eye drops were administered.

Twenty-four hours later, the pain resolved and IOP dropped to 16 mmHg in both eyes. The pupilsreturned to normal size and were fully reactive. A second gonioscopy showed angles open to scleral spur without peripheral anterior synechiae.

**Fig. 3:**
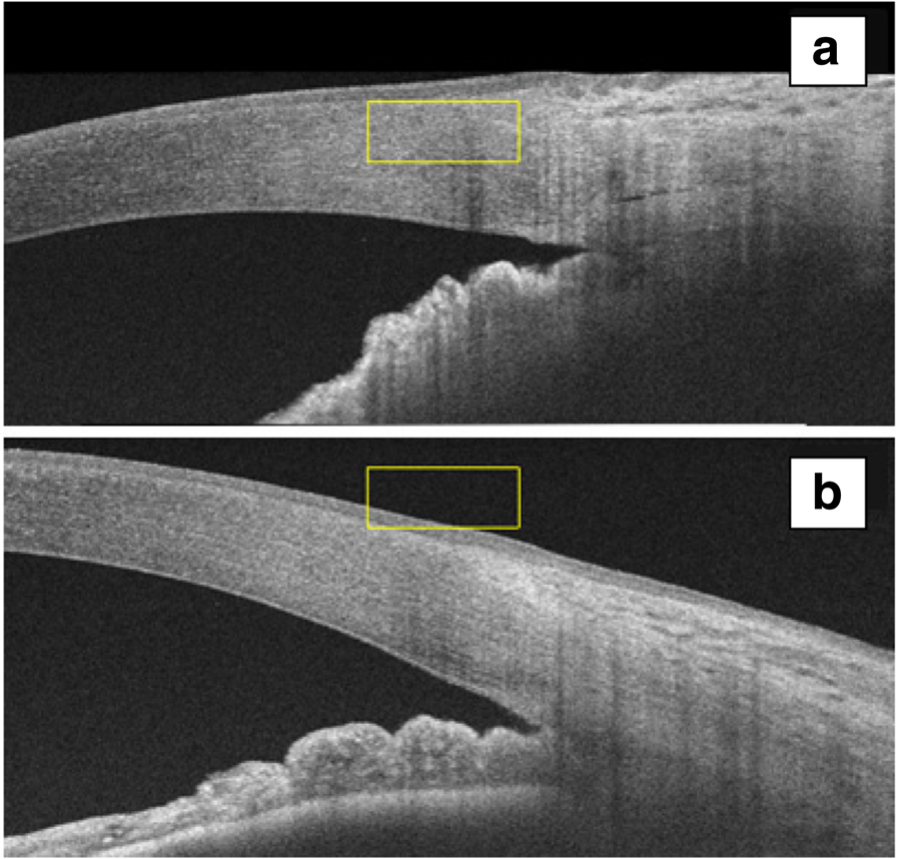
AS-OCT performed 72 h after Nd-YAG laser iridotomy shows angle opening in both right **(a)** and left **(b)** eyes

On day three of follow-up, the corneas became clearer, IOP remained well-controlled and the fundus examination showed a hyperhemic optic disc with normal C/D ratio. Subsequently NdYag laser iridotomy was successfully performed on both eyes (Fig. 3). The final visual acuity was 20/20 in both eyes and the intraocular pressure returned to normal levels.

## Discussion

Acute angle closure-glaucoma (AACG) is a painful, potentially blinding disease. Many systemic drugs may precipitate bilateral acute angle closure attacks. The most common of these drugs are the ⍺-adrenergic (Adrenaline, Ephedrine) and anticholinergic drugs (Atropine) through pupillary block mechanism following mydriasis in predisposed individuals with narrow iridocorneal angles [5]. Ocular conditions that predispose to angle closure are high hyperopia, shallow anterior chamber, and thick lens [6].

Other drugs may produce bilateral AACG secondary to ciliochoroidal effusion with anterior rotation of the ciliary body and forward displacement of the lens-iris diaphragm [7, 8]. They include mainly sulfonamide derivatives and Topiramate [2, 7].

To the best of our knowledge, the association of Tramadol administration with bilateral acute angle closure has not been previously reported. Tramadol is a commonly prescribed opioid used in severe pain. Tramadol can cause miosis through stimulation of opioid receptors [9] or mydriasis through stimulation of the adrenergic receptors [10]. In fact, it inhibits serotonin and noradrenaline reuptake from the synaptic cleft, and stimulates pre-synaptic release of serotonin [11]. Thus, the concentration of these two substances increases and so do their effects. The ubiquitous pupillary response to Tramadol may dependon individual metabolic abilities. In fact, miosis is more likely to occur in extensive Tramadol metabolisers while in intermediate and poor metabolisers, mydriasis might develop because of a delay in conversion of Tramadol to its active metabolite [12].

## Conclusion

Tramadol administration may cause mydriasis which can precipitate an attack of acute AACG in predisposed individuals with shallow anterior chambers. All clinicians should be aware of the potential risk of AACG in patients treated with Tramadol.

## Abbreviations

AS-OCT: Anterior segment optical coherence tomography
AACG: Acute angle-closure glaucoma
IOP: Intraocular pressure
LE: Left eye
RE: Right eye
